# Reirradiation of head and neck cancer focusing on hypofractionated stereotactic body radiation therapy

**DOI:** 10.1186/1748-717X-6-98

**Published:** 2011-08-21

**Authors:** Hideya Yamazaki, Naohiro Kodani, Mikio Ogita, Kengo Sato, Kengo Himei

**Affiliations:** 1 Department of Radiology, Graduate School of Medical Science, Kyoto Prefectural University of Medicine, 465 Kajiicho Kawaramachi Hirokoji, Kamigyo-ku, Kyoto 602-8566 Japan; 2 CyberKnife Center, Soseikai General Hospital, 126 Kami-Misu, Shimotoba Fushimi-ku, Kyoto Japan; 3 Radiotherapy Department, Fujimoto Hayasuzu Hospital, Hayasuzu 17-1, Miyakonojo, Miyazaki 885-0055, Japan; 4 Department of Therapeutic Radiology, Japanese Red Cross Medical Center, Hiroo, Shibuya-ku, 4-1-22, Tokyo, Japan; 5 Department of Radiology, Japanese Red cross Okayama Hospital, Aoe 2-1-1, Kita-ku, Okayama, Okayama Japan

**Keywords:** Head Neck cancer, reirradiation, Stereotactic radiotherapy, Bleeding

## Abstract

Reirradiation is a feasible option for patients who do not otherwise have treatment options available. Depending on the location and extent of the tumor, reirradiation may be accomplished with external beam radiotherapy, brachytherapy, radiosurgery, or intensity modulated radiation therapy (IMRT). Although there has been limited experience with hypofractionated stereotactic radiotherapy (hSRT), it may have the potential for curative or palliative treatment due to its advanced precision technology, particularly for limited small lesion. On the other hand, severe late adverse reactions are anticipated with reirradiation than with initial radiation therapy. The risk of severe late complications has been reported to be 20- 40% and is related to prior radiotherapy dose, primary site, retreatment radiotherapy dose, treatment volume, and technique. Early researchers have observed lethal bleeding in such patients up to a rate of 14%. Recently, similar rate of 10-15% was observed for fatal bleeding with use of modern hSRT like in case of carotid blowout syndrome. To determine the feasibility and efficacy of reirradiation using modern technology, we reviewed the pertinent literature. The potentially lethal side effects should be kept in mind when reirradiation by hSRT is considered for treatment, and efforts should be made to minimize the risk in any future investigations.

## Introduction

Locoregional failure is the predominant pattern of treatment failure and the most common cause of death in head and neck cancer patients [1]. As most recurrences occur in the first 2 years after primary treatment and 80% arise in previously high-dose irradiated volumes, reirradiation is a clinical challenge [2]. Chronic exposure of the upper aerodigestive tract to alcohol and tobacco, is the most common risk factor for head and neck cancer and is thought to produce field cancerization, a process in which patients are at risk for developing cancer at different mucosal sites. Second primary tumors in the head and neck can occur in up to 30% of patients within 10 years of onset [3-5]. The preference in operable patients is salvage surgery, with 5-year survival rates ranging from 16-36% [3,6,7]. However, due to tumor location and extent, medical contraindications, or patient refusal, surgery is often limited and compromised with close or positive margins, and only 20% of patients would undergo salvage surgery [3,7]. The major treatment has been palliative chemotherapy, which is associated with a median survival time (MST) of 5-9 months and response rates between 10-40% [3,8,9]. A few months of MST is generally anticipated for best supportive care [10]. High-dose reirradiation in inoperable patients is the only treatment option with any potential for cure. Reirradiation can be delivered using brachytherapy, stereotactic radiosurgery, or external beam radiotherapy with or without chemotherapy and with or without prior debulking surgery. Evidently, brachytherapy and stereotactic radiosurgery are attractive options for small-volume disease [11]. Several centers have reported encouraging results following aggressive reirradiation with or without chemotherapy. In contrast, reirradiation has caused severe adverse reactions in high-dose irradiated areas. We encountered nearly 10% lethal bleeding rate in our retrospective analysis of CyberKnife hSRT [12], in accordance with a recent report that cited 15% incidence of lethal bleeding after hSRT for carotid rupture syndrome [13]. Therefore, the aim of this article is to identify the possible prognostic and risk factors (particularly bleeding) for reirradiation, including stereotactic irradiation.

### Conventional radiotherapy (Table 1: additional file 1)

The earliest clinical studies of reirradiation were published in the 1980s and most were based on single institution experiences dating back to 1950 [14,15]. Repeat courses of radiation at 60 Gy, with total doses exceeding 120 Gy, were associated with severe complications; deaths as a result of bleeding were already observed in 5 (2 with necrosis) of 35 patients (14%) in one study and 2 of 85 patients with recurrent tumors (2.3%) in another study [14,15]. However, favorable clinical responses, including significant rates of sustained local disease control (25-60%), were observed [9].

Ohizumi et al. treated 44 patients of recurrent squamous cell carcinoma with cumulative dosing of more than 80 Gy [16]. The complete response rate was 32%. The median relapse-free survival time was 4 months, and the 5-year survival was 6%. They found that the anatomical location and an overlapping field of < 40 cm^2 ^were significant prognostic factors for survival. Favorable sites were the nasopharynx, larynx, and oropharynx; whereas, unfavorable sites were the oral cavity, nasal cavity, and hypopharynx. Severe late complications occurred in 5 (11%) patients.

De Crevoisier et al. reported the results of 169 patients with unresectable nonmetastatic head and neck cancers in a previously irradiated area [17]. Reirradiation protocols were as follows: radiotherapy alone (65 Gy over 6.5 weeks at 2 Gy/day) in 27 patients; Vokes protocol, i.e., 5-6 cycles of radiotherapy (median total dose, 60 Gy; 2 Gy/day) with simultaneous 5-fluorouracil (5-FU) and hydroxyurea in 106 patients; and bifractionated radiotherapy (median total dose, 60 Gy; 2 × 1.5 Gy/day) with concomitant mitomycin, 5-FU, and cisplatin in 36 patients. The median cumulative dose of the 2 irradiations was 120 Gy. Forty-four percent were local recurrences, 23% nodal recurrences, 14% both local and nodal recurrences, and 19% were second primary tumors. Mucositis grade 3 and 4 were observed in 32% and 14% of cases, respectively. Late toxicities (> 6 months) were as follows: cervical fibrosis (grade 2 to 3), 11%; mucosal necrosis, 21%; osteoradionecrosis, 8%; and trismus, 30%. Five patients died of carotid hemorrhage, apparently in complete remission. Thirty-seven percent of patients had complete responses. Patterns of failure were local only (53%), nodal only (20%), metastatic only (7%), and multiple (20%). The overall survival rate was 21% at 2 years and 9% at 5 years. The MST was 10 months for the entire population. Thirteen patients, of whom 12 were treated with the Vokes protocol, were long-term disease-free survivors. In a multivariate analysis, the volume of the second irradiation was the only factor significantly associated with the risk of death.

Salama et al. reviewed the University of Chicago experience with reirradiation in 115 patients from several chemoradiation trials [18]. Patients were treated with multiple 2-week cycles of 5 days of chemoradiotherapy, followed by a 9-day break. Radiotherapy was administered either daily or twice daily (bid), with a mean total dose of 64.8 Gy. The majority of patients were treated with computed tomography (CT)-based conformal radiotherapy. The MST was 11 months, and the 3-year overall survival rate was 22%. Approximately 41% of patients developed locoregional disease recurrence. Increasing the reirradiation dose, surgery before chemoradiation, and the use of cisplatin, paclitaxel, or gemcitabine were found to be significant predictors of improved survival. Toxicity was significant in 19 patients (17%) who died of treatment-related toxicity and 57% of patients who required a gastrostomy.

Spencer et al. reported results of the Radiation Therapy Oncology Group (RTOG) reirradiation trial (RTOG 96-10) conducted between 1996 and 1999 that included 81 patients [19]. All patients had unresectable squamous cell carcinomas and at least a 6-month interval between prior radiation and reirradiation (61 recurrences and 18 second primaries). The patients received a split-course regimen of radiation (total dose 60 Gy, 1.5 Gy fractions, bid) with concurrent 5-FU and hydroxyurea. Grade 3 to 4 mucositis occurred in 17% of patients, and lethal side effects occurred in 7.4% of patients (6/81). The 2-year overall survival rate was 16% (MST: 8.8 months). Survival among patients who had a longer interval between primary radiation and reirradiation was longer compared with those who had a shorter interval. Thereafter, Langer reported a succeeding RTOG 99-11 trial that employed hyperfractionated radiotherapy (1.5 Gy bid) for 2 weeks × 4 times with 2-week intervals, concomitant with daily cisplatin (15 mg/m^2^) and paclitaxel (20 mg/m^2^) during radiation, and granulocyte colony-stimulating factor during the off weeks [10]. The patients in that study had a 25.9% 2-year survival rate (MST: 12 months) and a mortality rate of 7.6% (8 patients). These results were an improvement over those observed in RTOG 96-10. Accordingly, to compare reirradiation and concurrent chemotherapy with chemotherapy alone in inoperable, previously irradiated, locally recurrent or second primary cancer, study RTOG 0421 (n = 240) was initiated. The concurrent reirradiation and chemotherapy arm of this trial used the same regimen as in RTOG 99-11; the chemotherapy-alone arm allowed selection of one of the 3 cisplatin-based chemotherapy regimens (cisplatin + paclitaxel, docetaxel, or 5-FU). The primary end point of this trial was survival. Unfortunately, this study ended prematurely because of inadequate accrual [20], presumably from a lack of provider interest in randomizing patients away from radiation treatment. Generally, the Western trials employed hyperfractionation radiotherapy on the basis of the assumption that lower single fractionation levels may reduce late adverse reactions. In addition, treatment strategies have used concurrent chemotherapy to overcome the decrease of radiation doses and possibly reduce metastatic failure.

Reentry McDonald et al. made a review that there were 41 reported Carotid blowout among 1554 patients receiving salvage reirradiation (2.6%); 76% were fatal [21]. In patients treated in a continuous course with 1.8-2-Gy daily fractions or 1.2-Gy twice daily fractions, 36% of whom received concurrent chemotherapy, the rate of carotid blowout was 1.3%, compared with 4.5% in patients treated with 1.5 Gy twice daily in alternating weeks or with delayed accelerated hyperfractionation, all of whom received concurrent chemotherapy (p = 0.002). There was no statistically significant difference in the rate of carotid blowout between patients treated with or without concurrent chemotherapy, or between patients treated with or without salvage surgery before reirradiation.

### Brachytherapy

Brachytherapy has achieved good local control in selected patients. Hepel reported of experiences in 30 patients reirradiated by high-dose-rate brachytherapy [22]. All of these patients were either inoperable, refused surgery, or had gross residual disease after salvage surgery for their recurrent disease. Thirty-six sites in these 30 patients were implanted by application of high-dose-rate interstitial brachytherapy technique with a tumor dose of 34 Gy (18-48 Gy) delivered by application of 300-400 cGy fractions, bid. Local tumor control was achieved in 69% of the implanted sites. Overall survival at 1 and 2 years was 56% and 37%, respectively. Grade 3 and 4 late complications occurred in 16% of the patients; however, no fatal complications were seen [22]. Although brachytherapy has a potential to cure oral, oropharyngeal, nasopharyngeal, and lymph node recurrences [20], only superficial small tumors can be treated, and the number of experienced institutions is limited.

### Intensity-Modulated Radiotherapy (IMRT)

A newer advancement in radiotherapy technique that facilitates precise dose delivery is the IMRT. This technique allows dose-escalation while minimizing normal tissue toxicity. Early reports suggest that IMRT can be used in an irradiation setting.

Sulman reported 58% 2-year survival rate and 64% local control rate by reirradiation using IMRT. Twenty percent of patients required admission and 1.4% suffered treatment-related deaths [20]. Twenty (27%) patients underwent salvage surgical resection and 36 (49%) received chemotherapy. The median reirradiation dose was 60 Gy and the median lifetime radiation dose was 116.1 Gy. Severe irradiation-related toxicity occurred in 15 patients (20%), and 1 treatment-related death was observed.

Popovitzer et al. reported on the appropriateness of limited-field irradiation for recurrent tumors. In an analysis of 66 cases of recurrence, only 2 recurred outside the irradiated area (4%); whereas, 50 recurred within the 95% isodose lines of the irradiated field, although a dose of 68 Gy was employed [23]. They concluded that a prophylactic field is not needed in reirradiation at present. The actuarial 2-year overall survival rate was 40%, and 71% of all patients developed a locoregional failure after 68 Gy of hyperfractionated radiotherapy. Mild to moderate late complications were common; 29% of patients experienced late morbidity of at least grade 3 or more. Two patients died of therapy-related complications (one cisplatin-induced renal failure, and one from aspiration). Two patients developed carotid artery blowouts, which were successfully salvaged.

Duprez et al. reported outcomes for 84 patients treated with IMRT (median dose, 69 Gy). Salvage surgery preceded reirradiation in 19 patients and 17 patients received concurrent chemotherapy [24]. Five-year locoregional control and overall survival were 40% and 20%, respectively. Stage T4, the time interval between initial treatment and reirradiation, and hypopharyngeal cancer were independent prognostic factors that showed worse overall survival from multivariate analysis. Twenty-six and 11 patients developed Grade 3 acute and late toxicity, respectively. No Grade 5 acute toxicity was observed. There were 2 fatal vascular ruptures during follow-up.

Biagioli et al evaluated their experience using every other- week IMRT with concurrent chemotherapy [25]. Patients who underwent surgery as a part of their salvage therapy had a mean estimated survival of 30.9 months compared with 22.8 months for patients who received only chemoradiotherapy (p = 0.126). Grade 3 or 4 acute toxicities occurred in 31.7% of patients, but all had resolved within 2 months of therapy completion. No deaths occurred during treatment, except for 1 patient, who died shortly after discontinuing treatment early because of previously undiagnosed metastatic disease; 6 patients had long-term complications. They concluded that concurrent chemotherapy with repeat radiotherapy with IMRT given every other week appears to be both well tolerated and feasible in patients treated with previous radiotherapy for recurrent head and neck cancer.

Lee et al. reported that patients who underwent IMRT, compared to those who did not, had a better 2-year LRPF (52% vs. 20%, p < 0.001) [26]. On multivariate analysis, non-nasopharynx and non-IMRT were associated with an increased risk of loco-regional (LR) failure. Patients with LR progression-free disease had better 2-year overall survival vs. those with LR failure (56% vs. 21%, p < 0.001). Acute and late Grade 3-4 toxicities were reported in 23% and 15% of patients. Severe Grade 3-4 late complications were observed in 12 patients, with a median time to development of 6 months after re-RT. They concluded that the use of IMRT predicted better LR tumor control

### Stereotactic radiosurgery (SRS)/Stereotactic radiotherapy (SRT) (Table 2: additional file 2)

With precise dose delivery, SRS/SRT has a physical advantage that allows highly conformal dose distribution and highly accurate dose-delivery to within a few mm for extracranial head and neck lesions [27]. The practical advantage is in terms of the short duration of treatment, generally lasting 1 day for SRS and approximately 2 weeks of alternate-day treatment for SRT. The lack of hematological or systemic toxicity permits inclusion of patients in poor general condition. Acute mucositis has been temporary and is well managed with supportive care. Siddiqui et al. treated 21 recurrent (n = 21) tumor patients with SRS/SRT [27]. Radiation doses were either single fractions of 13-18 Gy or 36-48 Gy in 5-8 fractions. The tumor control rate at 1 year was 60.6% and the MST was 6.7 months.

Treatment using the CyberKnife robotic system has several merits compared with γ-Knife treatment because the CyberKnife system can give a homogeneous dose distribution using fractionated image-guided radiotherapy, resulting in noninvasive fixation even for other than cranial lesions [28]. Roh et al. reported an 80% response rate (complete + partial) by with 30 Gy (range: 18-40 Gy) in 3-5 fractions by SRT using the CyberKnife system, which resulted in a 2-year survival rate of 30.9%. Late adverse reactions occurred in 8.6% of patients including 2.9% treatment-related deaths [29]. Other researchers have also achieved high efficacy using Cyberknife reirradiation, with response rates of over 70% and 2-year overall survival rates of 30% [28,30].

The University of Pittsburgh group initiated a phase I dose-escalation clinical trial [31]. Twenty-five patients were treated in 5 dose tiers with up to 44 Gy, administered in 5 fractions over a 2-week course. Neither grade 3/4 nor dose-limiting toxicities occurred. Four patients had Grade 1 or 2 acute toxicities. Four objective responses were observed for a response rate of 17%. Twelve patients had stable disease. The median time to disease progression was 4 months, and the MST was 6 months. Self-reported quality of life was not significantly affected by treatment. Fluorodeoxyglucose PET was a more sensitive early measure of response to treatment than CT volume changes. They concluded that reirradiation with up to 44 Gy using SBRT was well tolerated with no grade 4 or 5 treatment-related toxicities in the acute setting. Following this work, Rwigema et al. reported SRT outcomes in 85 patients using the CyberKnife system or Trilogy-IMRS that used a Varian stereotactic treatment-planning system [32]. The mean total dose of prior radiation to the primary site was 74 Gy (range: 32-170 Gy). Patients who were treated with ≥ 35 Gy had a significantly higher local control rate compared with those who received < 35 Gy (71% vs. 59%, respectively). This difference in local control rates was larger at tumor size greater than the median tumor volume (i.e., > 25 mL, 62% vs. 47%) compared with smaller volume disease (i.e., ≤ 25 mL, 80% vs. 71%). There were 34% complete responses and 34% partial responses; 20% of patients developed stable disease and 12% developed progressive disease. Among those with an initial tumor response followed by progression (58 patients), there was a median interval of 5.5 months for time to progression. The 1-year and 2-year local control and overall survival rates for all patients were 51.2% and 30.7%, and 48.5% and 16.1%, respectively. Overall, the MST for all patients was 11.5 months [32].

Georgetown university group reported feasibility of SRS/SRT reirradiation [33]. From 2002 to 2008, 65 patients received SRS/SRT and thirty-eight patients were treated definitively and 27 patients with metastatic disease and/or untreated local disease were treated palliatively. Nine patients underwent complete macroscopic resection before SRS/SRT. Thirty-three patients received concurrent chemoradiation. The median reirradiation SRS/SRT dose was 30 Gy (21-35 Gy) in 2-5 fractions. Fifty-six patients were evaluable for response: 30 (54%) had complete, 15 (27%) had partial, and 11 (20%) had no response. MST was 12 months. For definitively treated patients, the 2-year overall survival and locoregional control rates were 41% and 30%, respectively. Multivariate analysis demonstrated that surgical resection and nonsquamous histology were associated with improved survival. Seven patients (11%) experienced severe reirradiation-related toxicity, including one treatment-attributed death.

Kodani et al. evaluated the efficacy and safety of stereotactic body radiation therapy for patients with head and neck tumors [12]. Twenty-one patients were treated with CyberKnife SBRT. The prescribed dose ranged from 19.5 to 42 Gy (median, 30 Gy) in 3-8 fractions for consecutive days. The target volume ranged from 0.7 to 78.1 cm3 (median, 11.6 cm3). Treatment was well tolerated without significant acute complications in any cases. The overall survival rates was 50% at 24 months. The overall survival was better in patients without prior radiotherapy within the previous 24 months or in case of smaller target volume. Six patients suffered severe late complications, and 2 of them developed massive hemorrhage in the pharynx and both died of this complication 5 and 28 months, respectively..

### Site-specific consideration: Nasopharyngeal cancer (Table 3: additional file 3)

A substantial amount of data on reirradiation has been accumulated for nasopharyngeal carcinoma (NPC), although high evidence level data is not available [34-40]. Yu et al. reported on 275 patients from the Hong Kong Nasopharyngeal Study Group database who were evaluated for a first isolated local recurrence between 1996 and 2000 [34]. Two hundred patients received salvage treatment including external radiotherapy, brachytherapy, and/or surgery. The 3-year actuarial overall survival rate for patients with isolated local failure was 74%. On multivariate analysis, advanced initial T classification and the use of salvage treatment were independent prognostic factors. Symptomatic temporal lobe necrosis occurred in approximately 12% of patients and was the most morbid complication, with a mortality rate of 65%. As surgery can treat only limited small volume tumors, radiotherapy was used most often. However, there is little evidence for selection among the radiotherapeutic modalities. Treatment mode should be selected according to each patient's condition by determining age, performance status, tumor location, size, histology, past treatment, and the will of the patient.

Law et al. cited effectiveness of intracavitary mold brachytherapy in salvaging NPC with early-stage local persistence or first recurrence [35]. The overall complete remission rate was 97%. The rates of 5-year local control, relapse-free survival, disease-specific survival, overall survival, and major complication were 85%, 68.3%, 74.8%, 61.3%, and 46.9%, respectively. Major complications included nasopharyngeal necrosis with headache, necrosis of cervical vertebrae with atlantoaxial instability, temporal lobe necrosis, and palsy of the cranial nerves. The afterloaded mold was as effective as the preloaded version, but with fewer complications.

Wu et al. reported outcomes of SRT for 56 recurrent cancers from Tat-Sen University in Gantong, China [37]. They achieved a 3-year local progression-free survival rate of 75% by 48 Gy delivered in 6 fractions of SRT. Severe lethal adverse reactions were 2 bleeding episodes and 3 brain stem necroses.

Seo et al. reported good outcomes (23 complete responses, 5-year overall survival rate of 66%, local failure-free survival rate of 79%) for 35 nasopharyngeal cancer patients who received 33 (range: 24-45) Gy in 3-5 fractions by SRT using CyberKnife [38]. Favorable prognostic factors for overall survival were an early stage rT (recurrent T category, rT1-2: 80% vs. rT3-4: 39%) and age. Five patients showed adverse reactions of Grade 4-5.

Chua et al. designed a prognostic scoring system for radiosurgery [39]. A total of 48 patients with local failures of NPC were treated by stereotactic radiosurgery. The treatment was administered with a median dose of 12.5 Gy to the target periphery. The 5-year local failure-free probability after radiosurgery was 47.2% and the 5-year overall survival rate was 46.9%. Neuroendocrine complications occurred in 27% of patients but there were no treatment-related deaths. Five factors including age > 45 (age), time interval from primary radiotherapy > 6 months (time), rT4 disease (rT4), tumor volume ≥10 cc (tvol), and prior local failure (prior LF) were used to design the scoring system; the prognostic score = 0.22 × age (0, 1) + 0.27 × time interval > 6 months (0, 1) + 0.05 × rT4 (0, 1) + 0.28 ×Tvol (0, 1) + prior local recurrence (0, 1). Patients were then grouped by the calculated prognostic score: good prognostic group, 0 (i.e., those without any poor prognostic factors, n = 12), intermediate prognostic group, > 0 to 0.5 (n = 23), and poor prognostic group, > 0.5 (n = 13). The 5-year local failure-free probabilities in patients with good, intermediate, and poor prognostic scores were 100%, 42.5%, and 9.6%, respectively. The corresponding 5-year overall survival rates were 100%, 51.1%, and 0%, respectively.

Furthermore, Chua et al. also reported the superiority of SRT over SRS in a case control study [40]. They compared 43 cases of 12.5 Gy SRS and 43 cases of 34 Gy delivered in 2-6 fractions of SRT. The 1- and 3-year local failure-free survival rates were 70% and 51% for the SRS group and 91% and 83% for the SRT group (*P *= 0.003). Although the overall survival rates were similar (66% in SRS and 61% in SRT), severe side effects occurred at a rate of 33% for SRS (brain necrosis, 16%; bleeding, 5%), and 21% for SRT (brain necrosis, 12%; bleeding, 2%).

### Other lesions

Oropharyngeal and oral cancer have been good candidates for reirradiation treatments. For small recurrences in the larynx after conventional radiotherapy, Wang et al. reported excellent salvage outcomes by reirradiation in recurrent laryngeal cancer [41]. Single or a few lymph node recurrences also could be treated by reirradiation including brachytherapy or SRT [26,30,42]. Oral cancer can be salvaged by brachytherapy. However, upper gum, retromolar trigone, and palatal lesions were at a risk of fistula formation. Ogita et al. reported that prior surgical intervention or subcutaneous wide-spread tumor involvement were risk factors for post-radiotherapy refractory ulceration and bleeding [30]. There is no consensus on the usage of hSRT for larynx or hypopharynx, where swallowing movement may influence outcomes, since there is a general uncertainty in the homogeneity and reproducibility of dose distribution.

### Prognostic factors for survival outcome by reirradiation (Table 4: additional file 4)

#### Debulking surgery

De Crevoisier et al. reported the long-term results of salvage surgery prior to reirradiation in a small series of 25 patients [6]. In this series, patients who had positive margins and/or lymph node involvement with extracapsular extension following attempted salvage surgery were reirradiated with concomitant chemotherapy, consisting of 5-FU (800 mg/m^2^/day) and hydroxyurea (1.5 g/day). Radiation was delivered once daily for a total dose of 60 Gy in 2 fractions. Treatment was delivered as a 14-day cycle with 5 days of treatment followed by a 9-day break. These authors reported a 4-year survival of 43% and a 5-year disease-free survival of 26%. Surgical resection has the additional advantage of removing radioresistant disease. To address this, the Groupe d'Etude des Tumeurs de la Tete et du Cou and Groupe d'Oncologie Radiotherapie Tete et Cou groups in France jointly sponsored a phase III trial, which randomized 130 patients to surgery with or without adjuvant doses of 60 Gy and concurrent 5-FU/hydroxyurea. Progression-free survival was significantly improved in the adjuvant therapy arm, with an increase in acute and late complications. However, no overall survival benefit has been detected so far [43].

#### Tumor size rT1 to 3 vs. T4, T category/irradiated volume

Nonbulky tumors showed a trend toward improved tumor control, and patients with a low volume of disease prior to reirradiation were most likely to benefit from aggressive locoregional treatment [2,3,12,17,24,32,34,35,37-39,44]. In the same manner, smaller irradiated volume resulted in better outcomes, partly because a higher dosage of radiotherapy could be administered than that for a larger tumor [12,16].

#### Anatomical site

Among, highly selected patients, good outcomes were seen in patients with nasopharyngeal or laryngeal cancer in several studies [2,12,16,39-41], whereas hypopharyngeal cancer revealed poor prognosis [16].

#### Time interval since prior irradiation

Several studies including our own have reported the time interval to failure as an important prognostic factor [12,15,18,19,24,39]. Spencer et al. reported that the 1-year survival rate for patients treated within 3 years of prior radiotherapy was 35% compared with 48% for patients treated for > 3 years from prior radiotherapy, based on data from 81 patients (RTOG 96-10). Other patients who received their initial course of radiotherapy 24 months or more before the repeat course had a MST of 15 months vs. 6.5 months in patients who were treated within 1 year of their therapy [19].

#### Second primary versus recurrent tumor

As time to recurrence increases, it is more difficult to distinguish between a late recurrence and a second primary cancer; therefore, second primary have shown better outcomes than recurrence. New primary cancers should respond better to treatment than recurrent tumors in a previously irradiated field due to the inherent aggressiveness and radioresistance of recurrent tumor cells. Several studies have reported data, which support this hypothesis [11]. Based on data from the 81 patients treated in the RTOG 96-10 study, Spencer et al. reported that the 1-year survival rate and MST for patients with a second primary were 54% and 19.8 months, respectively compared with 38% and 7.7 months, respectively for patients with recurrent cancers [19]. Stevens et al. analyzed 100 patients treated with reirradiation alone and reported a 5-year actuarial overall survival and locoregional control of 17% and 27%, respectively for recurrent tumors compared with 37% and 60%, respectively for second primary cancers in a previously irradiated field [15].

#### Dose-response

Several reports have cited dose as a prognostic factor in reirradiated tumors [26,33]. The requirement of high-dose irradiation is because radiation-resistant clonogens could be a source of recurrence. In general, it is hard to imagine that smaller doses than used in the original irradiation treatment will be curative. Actually, despite aggressive therapy with high doses of reirradiation concomitant with chemotherapy, the majority of failures are still locoregional, illustrating the high proportion of radioresistant cells in recurrent tumors [23]. The University of Chicago found that the median and 2-year survival for patients receiving 58 Gy or more were 11.3 months and 35%, respectively compared with 6.5 months and 8%, respectively for patients receiving a lesser dose [18]. Also in an SRT series, a prescribed dose of 35 Gy or more and tumor volume of 25 mL or less had a better local control rate [32].

### Risk factors for adverse events (Table 4: additional file 4)

It can generally be stated that the incidence of high-grade toxicity associated with reirradiation is substantial. The risk of severe late complications was reported as 20-40% and was related to prior radiotherapy dose, primary site, retreatment radiotherapy dose, treatment volume, and technique. Although the reported frequency of high-grade acute toxicities varies greatly from study to study, less severe adverse effects such as mucositis and dermatitis have been universally reported to occur in majority of patients. The incidence of grade 3 to 4 mucositis, including dysphasia requiring a feeding tube or gastrostomy, has been generally reported to occur in 10- 40% of patients [19]. Grade 3 dermatitis has been observed in < 10% of patients. The frequency of significant hematological toxicity varies considerably in the literature and appears to depend largely on the chemotherapy regimen used. Ulcer formation has been frequently found in cases with histories of previous surgical intervention or mucosal involvement. Ogita et al. reported that ulcer formation occurred in 53.2% of cases with mucosal involvement vs. 30.7% without mucosal involvement using CyberKnife SRT at 1 year after treatment [30]. Other chronic adverse reactions that occurred were as follows: cranial nerve palsies, brain necrosis (temporal lobe, etc.), osteoradionecrosis (skull base, mandibular, etc.), palatal fibrosis, trismus, aspiration, hormonal dysfunction, headache, otitis media and hearing impairment, corneal ulcer, retinopathy, cerebrospinal fluid leakage, brain herniation, and radiation induced malignancy.

Life-threatening toxicities caused by reirradiation occur infrequently; however, by nature they are quite worrisome. Carotid rupture in the setting of reirradiation in nearly all instances results in death [12,13]. De Crevoisier et al. reported 5 such cases, the University of Chicago reported 6 cases (1 where the patient survived), and 2 cases were reported in RTOG 99-11 [10]. Generally, those treated by conventionally fractionated reirradiation reported bleeding rates of 3-14%, and a recent IMRT series reported bleeding rates of 0-3% [4,14,21,24]. On the other hand, several SRS and hSRT series have reported higher rates (9-15%) of bleeding, including CyberKnife hSRT [12,13,36]. Cengiz et al. reported a high incidence (15%) of bleeding after SRT by the Cyberknife system [13], which is similar to our experiences of bleeding rates (9.5%). These studies reported that this fatal syndrome occurred only in patients with tumors surrounding carotid arteries and where the carotid arteries received all of the prescribed dose. Necrosis frequently appears before bleeding, and it has been difficult to make a differential diagnosis of recurrence with or without infection [12,13,36]. Xiao et al. described several considerations regarding bleeding after reirradiation for NPC based on their experience with 8 cases of bleeding. For a tumor involving the Rosenmueller fossa that invades deeply into the foramen lacerum, which is the location where the cervical portion of the internal carotid artery curves upward and enters the cranium is problematic as it is very near the Rosenmueller fossa [36]. This anatomical site is quite vulnerable to hemorrhage, particularly, when the tumor has not only surrounded the arterial wall but has also invaded and damaged it. Furthermore, when the artery has been weakened and affected by infection and necrosis, copious bleeding easily occurs. Bleeding may also be caused by due to the following: a total high local dose resulting from a second course of external irradiation, complicating diabetes mellitus, and a single dose that is too high (1 patient received 15 Gy in one fraction plus 12 Gy in another fraction over 12 days). After this patient's death, the single dose was reduced for subsequent patients.

## Discussion

Outcomes after reirradiation of tumors are variable, and 5-year survival rates range from as low as 3.8% in unselected patients to as high as 100% in selected patients [19,39]. We could not find any randomized prospective trial to determine the best radiotherapy schedule and modality. The interpretation of the small number of prospective and the many retrospective studies including inhomogeneous patient characteristics was inconclusive. Several complicating factors included variety of patients with recurrent and second primary tumors, limited and advanced tumors, curative and palliative treatment, squamous cell carcinoma and other histologies, and variable treatment strategies. Several studies have demonstrated that reirradiation is a feasible option in previously irradiated head and neck cancer patients. As noted above, the treatment options for these patients are limited. Several studies have used different inclusion criteria for reirradiation. The RTOG trials were somewhat more stringent than other series and in that at least 75% of the irradiated tumor volume had to be previously treated with at least 45 Gy. Other studies classified patients with any overlap between initial and salvage treatment as having been reirradiated [16,20]. As discussed, patients with resectable disease frequently enjoy improved salvage rates. Such patients are frequently offered adjuvant reirradiation alone or with chemotherapy. The likelihood of cure is impacted by the interval between the initial course of radiotherapy and reirradiation depending on, whether the carcinoma is a recurrence or a second primary tumor, initial T-stage (rT stage), whether the tumor is isolated or local-regional, and the histology (Table 4: additional file 4) [11].

A practical advantage of hSRT is the shorter duration of treatment, with SRS generally lasting 1 day and alternate-day hSRT lasting approximately for 1 to 2 weeks. Also, the lack of hematological or systemic toxicity permits the inclusion of patients in poor general condition. Acute mucositis has been temporary and well managed with supportive care. The physical advantage of stereotactic radiation arises from the ability to achieve a highly conformal dose distribution and deliver the treatment with high accuracy. Several limitations should be considered in such advanced limited field radiotherapy. Contour delineation is a problem to be resolved, especially in multi-institution trials. There is a wide range of deviation in GTV, CTV, and PTV delineation methods and the prescribed methods are varied (D50 to D95), depending largely on the physician's decision, all of which become more important if employed for a limited small field [45].

The biological effective dose (BED) formula has not been established for treatment effectiveness of hSRT because of the lack of experimental validity when large doses per fraction and short overall treatment times have been used. However, the BED formula currently serves as a useful model for biological comparison of different fractionations, particularly for adverse reactions. For estimation of late complications, King et al. made a consideration for rectal mucosal side effects in prostate cancer radiotherapy. They gave 5 × 7.25 Gy = 36.25 Gy for prostate stereotactic body radiotherapy and reported that a reduced rate of severe rectal toxicity was observed with treatment every other day vs. treatment over 5 consecutive days (0% vs. 38%, *P *= 0.0035), although none was as high as Grade 3. They predicted that the acute equivalent total dose in 2-Gy fractions was 52.1 Gy for treatment with "daily" fractions (5 fractions per week) but only 50.8 Gy for treatment every other day [46]. In addition, the University of Pittsburgh group was able to escalate the dose up to 44 Gy in 5 fractions without any carotid blow out syndrome using daily protocol, even though follow-up periods were short [31]. They used every-other-day (QOD) hSRT which may contain the potential impact on adverse toxicities because most of the papers in hSRT that have seen high incidences of carotid blow-out for example used a once-daily (QD) hSRT approach.

IMRT can optimize the treatment plan and more easily spare critical structures thus reducing adverse reactions. These benefits have been proven in various fields and may also apply to reirradiation patients. Therefore, higher risk patients, such as those in whom the tumor involves more than half the circumference of the carotid artery, might be better candidates for treatment by IMRT with conventional fractionation. In contrast, some investigators argue that the dose inhomogeneity and inaccurate delivery to tumor lesions noted in inverse planning can lead to inadequate target coverage. To overcome the problems of precise location and changing shape of the tumor during treatment periods, image-guided radiotherapy and adaptive radiotherapy were introduced in several institutions. In addition, we anticipate that the ability of IMRT to carefully sculpt the dose around critical structures and thus increase the total dose, will outweigh the theoretical concerns of an increased volume of tissue receiving low-dose radiation. However, the availability of IMRT is limited, and some patients cannot endure long radiotherapy schedules of 5-6 weeks or more. For patient convenience, hSRT should be explored further.

One of the future trends is development of drugs such as EGFR inhibitors that have improved outcomes in head and neck cancer treatment [47]. A recent European randomized trial showed that addition of cetuximab, the first clinically available EGFR-directed monoclonal antibody, to a standard chemotherapy regimen (platinum/5-FU) led to an improved survival benefit. This study, with support from the results of an additional smaller study in the US, has changed practice [47]. Accordingly, additional EGFR blockade trials in reirradiation are ongoing in several institutions [48,49]. Heron et al reported the result of phase II study (a single institution matched case-control study) that cetuximab conferred an overall survival advantage (24.5 vs. 14.8 months) when compared with the stereotactic body radiotherapy alone arm, without a significant increase in grade 3/4 toxicities [49].

In conclusion, reirradiation treatment is a challenging field but is feasible and potentially beneficial for patients who otherwise may not be salvaged by other available options. Future investigation is warranted but should include careful patient selection with consideration of the radiotherapy schedule, tumor factors as well as patient history and characteristics.

## Competing interests

The authors declare that they have no competing interests.

## Authors' contributions

HY, NK: conception and design. HY drafted the manuscript, MO, KS, and KH criticized the manuscript. All authors read and approved the final manuscript.

## Supplementary Material

Additional file 1 **Table 1**. Re-irradiation for Head and Neck cancer (various sites) Title: A title to explain what is in the file. Click here for file

Additional file 2 **Table 2**. Re-irradiation using stereotactic irradiation. Click here for file

Additional file 3 **Table 3**. Reirradiation for nasopharyngeal cancer. Click here for file

Additional file 4 **Table 4**. Reported Prognostic factors and Risk factors. Click here for file
